# Types and Effectiveness of Community-Based Cardiovascular Disease Preventive Interventions in Reducing Alcohol Consumption: A Systematic Review and Meta-Analysis

**DOI:** 10.7759/cureus.61323

**Published:** 2024-05-29

**Authors:** Neamin M Berhe, Hamid Y Hassen, Jean-Pierre Van Geertruyden, Rawlance Ndejjo, Geofrey Musinguzi, Hilde Bastiaens, Steven Abrams

**Affiliations:** 1 Department of Primary & Integrated Care, Department of Family Medicine and Population Health, Faculty of Medicine and Health Sciences, University of Antwerp, Antwerp, BEL; 2 Department of Health Sciences, Société Générale de Surveillance (SGS) Life Sciences, Mechelen, BEL; 3 Global Health Institute, Department of Family Medicine and Population Health, Faculty of Medicine and Health Sciences, University of Antwerp, Antwerp, BEL; 4 Department of Disease Control and Environmental Health, School of Public Health, Makerere University, Kampala, UGA; 5 Data Science Institute, Interuniversity Institute for Biostatistics and Statistical Bioinformatics, Hasselt University, Diepenbeek, BEL

**Keywords:** systematic review and meta-analysis, prevention, alcohol consumption, effectiveness, community-based intervention, cardiovascular disease

## Abstract

Cardiovascular disease (CVD) poses a global health challenge, with modifiable risk factors, notably alcohol consumption, impacting its onset and progression. This review synthesizes evidence on the types and effectiveness of community-based interventions (CBIs) aimed at reducing alcohol consumption for CVD prevention. Electronic databases were systematically searched until October 31, 2019, with updates until February 28, 2023. Given the heterogeneity in outcome measures, we narratively synthesized the effectiveness of CBIs, adhering to the synthesis without meta-analysis (SWiM) guidelines for transparent reporting. For selected homogenous studies, a random-effects meta-analysis was utilized to estimate the effects of CBIs on alcohol consumption. Twenty-two eligible studies were included, with 16 demonstrating that CBIs reduced alcohol consumption compared to controls. Meta-analysis findings revealed reductions in above moderate-level alcohol consumption (pooled odds ratio (OR)=0.50, 95% confidence interval (CI): 0.37, 0.68), number of alcohol drinks per week (standardized mean difference=-0.08, 95% CI: -0.14, -0.03), and increased odds of low-risk drinking (pooled OR=1.99, 95% CI: 1.04, 3.81) compared to the control groups. Multi-component interventions (particularly those combining health education, awareness, and promotion activities) and those interventions with a duration of 12 months or more were notably effective. The beneficial effects of CBIs focusing on achieving a reduction in alcohol consumption showed promising outcomes. Implementing such interventions, especially multicomponent interventions, could play a significant role in mitigating the increasing burden of CVDs. Future studies should also consider employing standardized and validated tools to measure alcohol consumption outcomes to enhance the consistency and comparability of findings.

## Introduction and background

Cardiovascular diseases (CVDs) impose a huge socio-economic burden on communities and the health system. In the last three decades, the global prevalence of CVDs has nearly doubled from 271 million in 1990 to 523 million in 2019, while CVD-related mortality has increased by more than 50% from 12.1 million in 1990 to 18.6 million in 2019 [1]. Over the same period, years lived with disability due to CVDs has doubled from 17.7 million in 1990 to 34.4 million in 2019 [2]. Consequently, CVDs have become the largest single contributor for noncommunicable diseases accounting for one-third of the annual deaths across the world [1,3]. An increase in age-standardized CVD rate has been observed in countries that were once known to have a declining trend [2]. The morbidity and mortality vary between countries and regions due to the influence of culture, globalization, industrialization, epidemiological and demographic transition, and the prevalence of other risk factors [4,5]. More precisely, the prevalence of modifiable risk factors, including excessive alcohol use, is known to contribute to the burden of CVDs [2].

Although the link between excessive alcohol consumption and CVD has long been recognized, more recent evidence is challenging the notion of any beneficial effects related to moderate alcohol consumption [3,6,7]. A recent World Heart Federation and World Health Organization (WHO) report indicated that even small amounts of alcohol consumption raise the risk of CVDs, including coronary disease, stroke, heart failure, cardiomyopathy, atrial fibrillation, and aneurysm among adults [8,9]. In light of this evidence, targeting alcohol consumption through the development, implementation, and evaluation of cost-effective interventions has been prioritized [10].

One of the promising cost-effective strategies focusing on a population-level reduction of CVDs is the implementation of community-based interventions (CBIs) [11-13]. CBIs for CVD prevention aim to reduce the CVD burden by targeting major modifiable risk factors of CVDs, including excessive alcohol consumption, by focusing on the entire community rather than only on high-risk individuals in healthcare settings [14].

A few reviews have explored the effectiveness of community-based CVD interventions in reducing alcohol consumption. However, these reviews were limited in scope, focusing on specific contexts and populations. Portheet al. reviewed eight randomized controlled trials and quasi-experimental and time-series studies conducted in high-income countries and identified CBIs to be effective in reducing alcohol consumption [15]. In contrast, Ndejjo et al. reviewed studies in low-middle-income countries and found mixed results regarding the effectiveness of interventions in improving alcohol consumption [16]. Thus, these reviews were not comprehensive and reported inconclusive findings, highlighting the need for a systematic review and synthesis of available evidence in scientific literature.

To address these gaps, this review aimed to synthesize comprehensive evidence on the types and effectiveness of CBIs for CVD prevention targeting the reduction of alcohol consumption. The findings from this review provide important insights for policy-makers and public health practitioners to bolster CBIs for CVD prevention in the context of alcohol consumption.

## Review

Methods

This review is part of a multi-country CVD prevention project named SPICES - Scaling-up Packages of Interventions for CVDs in selected sites in Europe and Sub-Saharan Africa (https://www.uantwerpen.be/en/projects/spices/). One of the aims was to review the available evidence on community-based CVD preventive interventions targeting CVD risk factors and knowledge. This specific review focused on studies targeting the reduction of alcohol consumption as one of the outcomes. The review protocol is available in the PROSPERO International (Prospective Register of Systematic Reviews; registration number: CRD42019119885). To ensure standard and complete reporting of this review, the Preferred Reporting Items for Systematic Reviews and Meta-Analyses (PRISMA) 2020 guidelines were complied with (https://www.prisma-statement.org/prisma-2020).

Study Selection

Studies were included in this review if they focus on CVD prevention and targeted reduction of alcohol consumption as an outcome. The following are the criteria for including studies in this review:

Population: Studies were included if they involved (adults above 18 years old) who were not diagnosed with any type of CVD upon study enrolment, regardless of gender.

Intervention: Studies that evaluated interventions for CVD prevention; were based or implemented in the community using simple or multi-component delivery strategies; conducted in, but not limited to, religious centers, schools, households, pharmacies, and primary healthcare units; focusing on either primordial or primary prevention; and aimed at targeting risk factors associated with any CVDs.

Comparator: Studies were included if comparators included usual care, standard general practitioner (GP) referral, enhanced usual care (EUC), or waiting-list controls.

Outcome: Studies were only included if they reported outcomes relevant to alcohol consumption, whether data were obtained through self-reporting, self-administered questionnaires, or interviews.

Study designs: Studies that employed individual or clustered randomized controlled trials, or controlled quasi-experimental, or interrupted time series studies were eligible.

Studies that evaluated interventions involving clinical procedures, pharmacologic components, or solely took place in clinical settings were excluded. Furthermore, studies with a follow-up duration of less than nine months, an attrition rate above 40%, or a total sample size below 150 were excluded. Studies that were reported in the English language were considered, but there was no limitation in terms of study location.

Search Strategy

International electronic databases, including Medline, Embase, CINAHL, Cochrane Register of Controlled Studies, and PsycINFO, were searched until October 31, 2019. To include recent relevant studies, the search was updated until February 28, 2023. Other sources, including thesis online, OpenGrey, ProQuest, CHW Central, Google Scholar, ClinicalTrials.gov, and the WHO International Clinical Trials Registry Platform were also searched for relevant similar articles. Based on a preliminary keyword search, a systematic search strategy was developed using terms related to population, intervention, and outcomes. Details of the search strategy are available in a previous publication [17], and the search terms in Medline are available in the supplementary materials (Box S1). Citation mining was also done by reviewing the reference list of the included articles.

Selection Process

**Figure 1:**
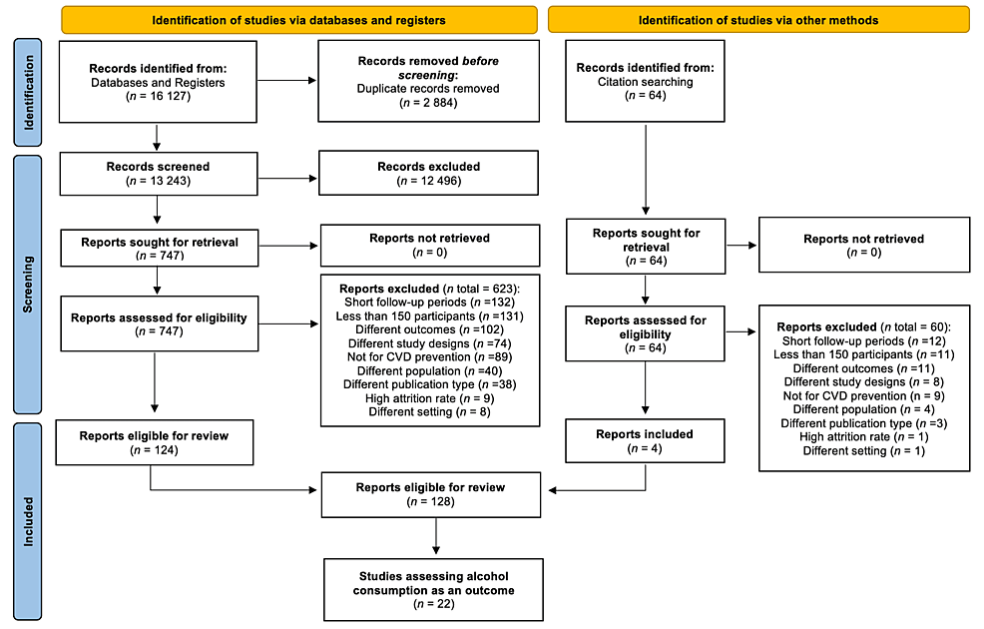
Illustration of the article selection process using the PRISMA flow chart. CVD, Cardiovascular disease; PRISMA, Preferred Reporting Items for Systematic reviews, and Meta-Analyses

Articles retrieved from electronic databases were exported as a single library using EndNote and were then verified and deduplicated. Subsequently, deduplicated searches were imported into Rayyan.ai software (http://rayyan.qcri.org/). Three reviewers (HYH, RN, and NMB) independently screened all articles by reviewing their titles and abstracts, using predefined inclusion criteria to determine whether each article met the requirements for inclusion in the review. Moreover, articles that were included in the full-text screening were assessed by two reviewers (HYH and NMB) for eligibility to be included in the review. When decisional conflicts arose regarding the inclusion or exclusion of an article and a final decision could not be reached through consensus, an arbitrator (RN) was designated to resolve the conflict and make the final decision. For full-text articles with missing or incomplete information, the corresponding author(s) were emailed twice. Justifications for excluding studies during the full-text process were documented and presented in the PRISMA flow chart (Figure 1).

Risk of Bias Assessment

Quality appraisal of evidence for the included randomized controlled trials was assessed following the revised Cochrane tool for Risk of Bias (RoB2) [18]. This tool was also used to appraise cluster-randomized controlled trials by evaluating additional domains that account for bias due to clustering. The risk of bias in non-randomized studies was assessed using the Risk of Bias in Non-Randomized Studies of Interventions (ROBINS-I) tool [19]. All studies’ risk of bias were independently assessed by two reviewers (HYH and NMB).

Data Extraction

Data extraction was performed by two independent reviewers (NMB and HYH), following the development of the data extraction forms. All disagreements between authors were resolved by consensus or arbitrated by a third person, if necessary. Study characteristics, including study population, intervention and its description, comparators, outcomes and outcome measures, intervention duration, study design, study settings, methodological approach (e.g., measurement tools and statistical analysis used), sample size, attrition rate, results, and funding sources, were extracted rigorously. In addition, effect estimates with 95% confidence intervals (CIs) and the direction of effects were extracted and validated after data extraction. In a few studies where results were presented solely using graphs, we utilized WebPlotDigitizer [20] only after a failed attempt to contact the study authors. 

Data Synthesis

Due to the heterogeneity in the outcome measures used, evidence for the effectiveness of CBIs for CVD prevention was predominantly narratively synthesized for all the included studies. To enable transparent reporting, the Synthesis Without Meta-analysis (SWiM) guideline was utilized to present our findings [21]. The nine-item SWiM checklist is available in the supplementary material (Appendix, Table S5). We grouped studies using study design, target population, and intervention types. Data are presented in tables using information related to the country, year of publication, type of study design, intervention type and duration, target population, setting, measures of alcohol consumption, and risk of bias assessment of included studies. Results are presented and discussed in relation to the income per capita classification of countries (high-income countries (HICs) vs. low- and middle-income countries (LMICs)), target population, risk of bias, measures of alcohol consumption, type of study design, intervention setting, and duration. Mean differences, odds ratios, and adjusted regression coefficients were used to compare study groups and present findings from the included studies. Finally, to synthesize the overall evidence, vote counting based on the direction of effect was used.

Meta-Analysis

Studies that reported similar study populations and measures of alcohol consumption were synthesized using a meta-analysis. For continuous outcome measures, standardized mean differences (SMD) with 95%CIs were presented, while for dichotomous outcomes, the strength of association was expressed in terms of odds ratios (ORs) with 95%CIs to provide the pooled effect estimate. When standard deviations and/or standard errors were not reported in the original studies, these quantities were imputed using other reported parameters based on the Cochrane guideline. For studies that reported multiple intervention or control arms, groups were combined to conduct a single pair-wise comparison [22]. This decision was based on the arms being sufficiently similar in terms of delivery methods, outcome measures, participants' characteristics, and the setting and duration of the study. Random effects meta-analyses were used to account for between-study variability across the included studies. Lastly, heterogeneity was assessed using the I^2^ statistic, and its significance was tested using the Q statistic [23].

Results

We identified 16,118 titles/abstracts from databases and 64 from manual searches. After screening for duplicates and titles/abstracts, we reviewed 817 full-text articles. Out of these, 128 studies fulfilled the eligibility criteria, with 22 of them reporting on at least one measure of alcohol consumption as an outcome. Among the 22 included studies, eight were considered in the meta-analysis (Figure 1).

Study Characteristics

Twelve of the 22 studies were from HICs, including Japan (n=3) [24-26], the United States (n=2) [27,28], Canada (n=2) [29,30], Spain (n=1) [31], Denmark (n=1) [32], Sweden (n=1) [33], the United Kingdom (n=1) [34], and the Netherlands (n=1) [35]. Ten studies were conducted in LMICs, including China (n=4) [36-39], Sri Lanka (n=2) [40,41] Kenya (n=2) [42,43], India (n=1) [44], and Vietnam (n=1) [45] (Table 1, Appendix Table S4).

**Table 1:** Study characteristics of the included articles. NI, Not indicated; MA, Included in the meta-analysis

Author, Year	Country	Intervention Duration (Months)	Intervention Setting	Participant Age Range or Mean (SD)	Sample Size	
					Intervention group (s)	Control group
Individual randomized studies						
Crombie et al., 2018 [34]	United Kingdom	3	Home-based	25-44	411	414
Hansen et al., 2012 [32]	Denmark	6	Community-based	49-66	706	358
Lu et al., 2015 [37]	China	24	Community-based	40-75	231	116
Sobell et al., 2002 [30] ^MA^	Canada	4-8	Home-based	18+	321	326
Takahashi et al., 2006 [25]	Japan	2	Community-based	40-69	224	224
Zhang et al., 2018 [39]	China	24	Primary healthcare setting	60+	323	314
Chum et al., 2020 [29]	Canada	24	Community-based	18+	256	196
Okube et al., 2022 [42]^MA^	Kenya	12	Community-based	18-64	156	138
Clustered randomized studies						
Boveda-Fontan et al., 2015 [31^MA^	Spain	12	Primary healthcare setting	40-75	107	120
Chandraratne et al., 2019 [40]^MA^	Sri Lanka	12	Community-based	Intervention group-46.1(8.1), Control group-44.8(8.2) (adults)	262	250
Ettner et al., 2014 [27]^MA^	United States	NI*	Primary healthcare setting	60+	546	640
Fink et al., 2005 [28]^MA^	United States	30	Primary healthcare setting	65+	443	222
Siriwardhana et al., 2013 [41]^MA^	Sri Lanka	3	Community-based	18-80	103	99
Thankappan et al., 2018 [44]	India	12	Community-based	30-60	500	507
Wang et al., 2020 [13]	China	24	Community-based	18-60	3178	988
Non-randomized studies						
Haruyama et al., 2009 [24]	Japan	6	Community-based	65+	232	204
Huang et al., 2011 [36]^MA^	China	36	Community-based	35+	826	806
Kloek et al., 2006 [35]	Netherlands	24	Community-based	18-65	1426	1355
Nguyen et al., 2007 [45]	Vietnam	36	Community-based	25+	1185	1190
van de Vijver et al., 2016 [43]	Kenya	6	Community-based	35+	1531	1233
Zhu et al., 2013 [26]	Japan	6	Community-based	40-74	347	1636
Törmä et al., 2021 [33]	Sweden	NI*	Community-based	40+	2555	2845

**Figure 2:**
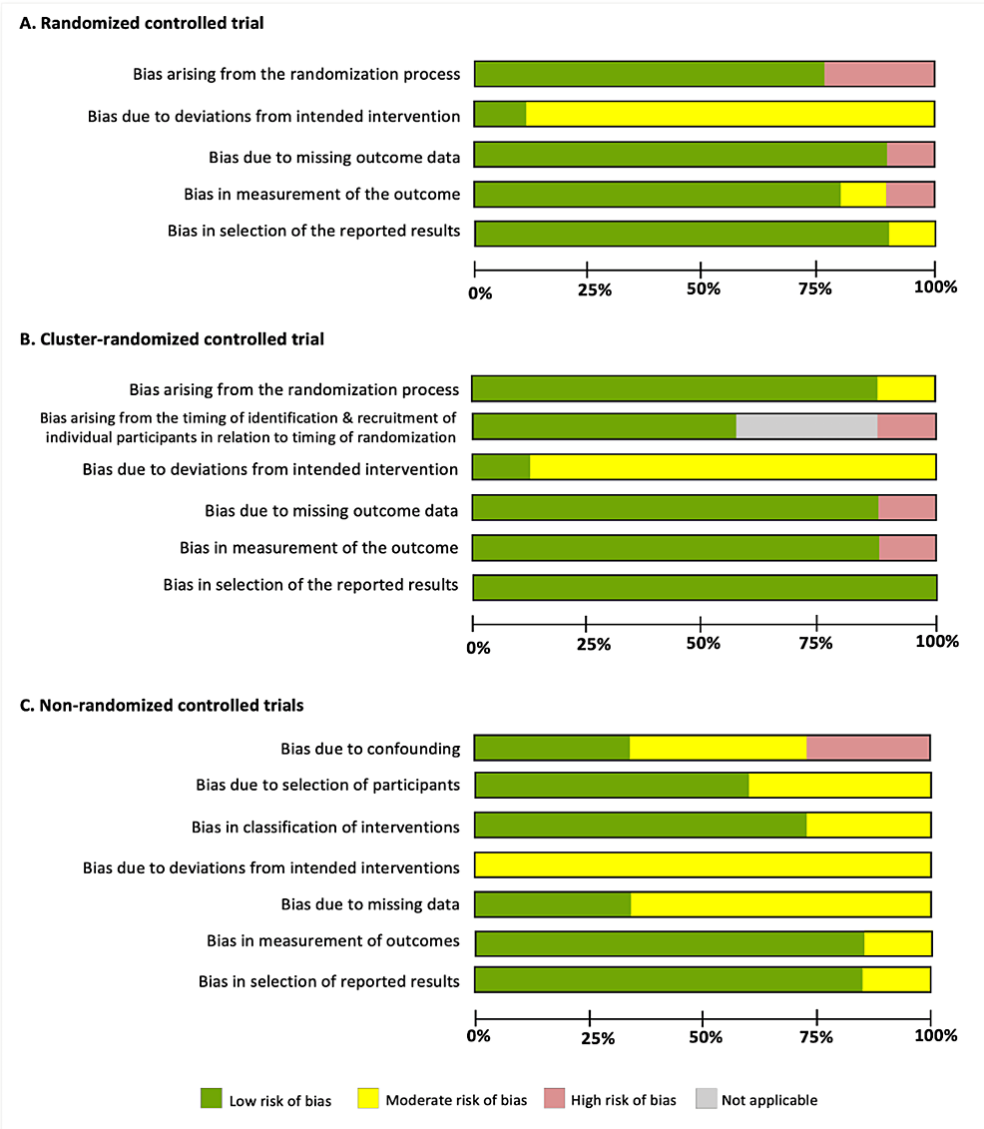
Risk of bias assessment of the included studies for all domains.

**Figure 3:**
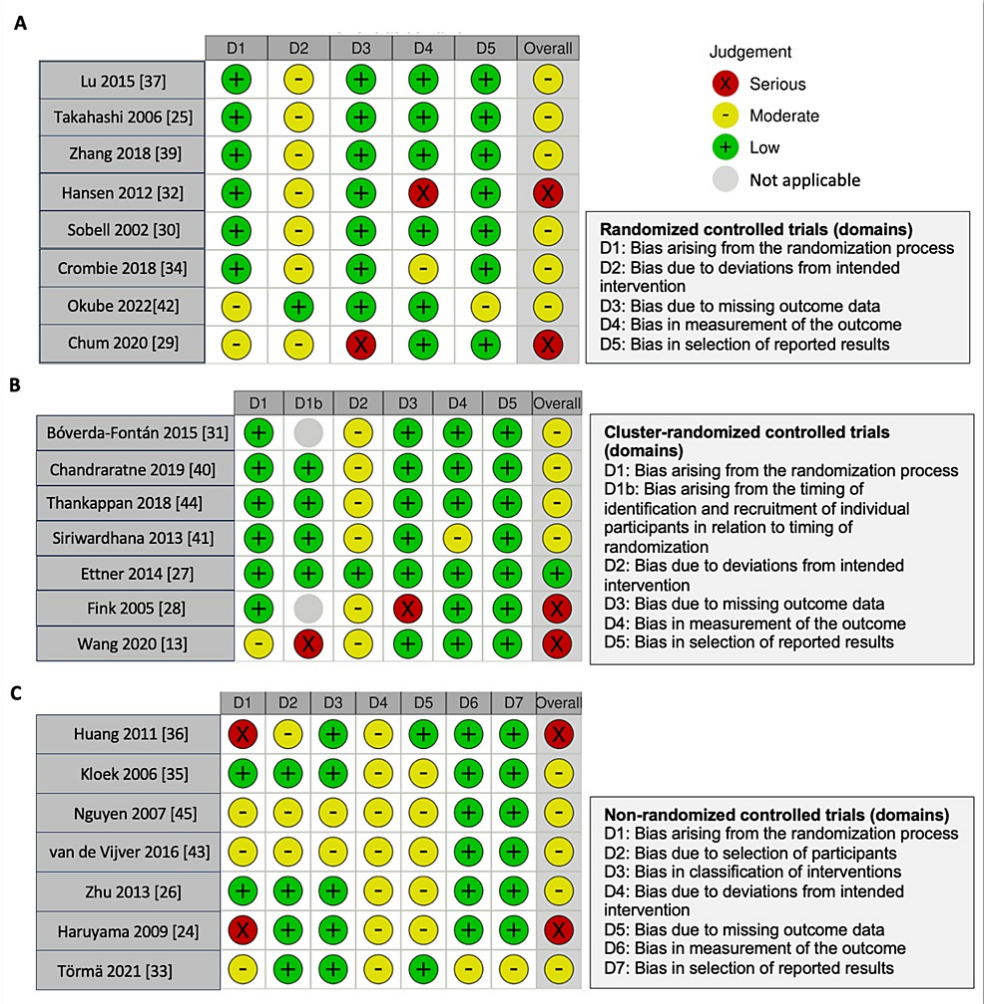
Detailed risk of bias assessment for each included study with (A) randomized controlled trial, (B) cluster-randomized controlled trial, and (C) non-randomized controlled study design.

Of the 22 studies reviewed, 15 were randomized, including eight individual randomized trials (RCTs) [25,29,30,32,34,37,39,42] and seven cluster randomized trials (CRCTs) [27,28,31,38,40,41,44]. The remaining seven were non-randomized controlled studies (NRCs) [24,26,33,35,36,43,45]. Risk of bias assessments revealed that two RCTs had high risk due to outcome measurement or data handling [29,32], while two CRCTs faced high risk related to recruitment and randomization timing or data handling [28,38]. Among the NRCs, two had a high risk of bias due to confounding [24,36] (Figures 2, 3; Appendix Figure S1).

Measures of Alcohol Consumption 

Studies used various methods to measure alcohol consumption. Thirteen studies reported continuous outcome measures, such as number of drinks per week [27,28,30-32], number of drinks per day or per drinking occasion [30,35,42,44], number of days alcohol was consumed to intoxication in the past 30 days [29], percentages of energy from alcohol [33], and daily grams of alcohol consumed [25,37]. Among 18 studies that reported categorical outcome measures, four utilized validated instruments to identify levels of risky drinking such as the Alcohol Use Disorder Identification Test (AUDIT) [34,41], Computerized Alcohol Related Problem Scoring (CARPS) [28], and the Comorbidity Alcohol Risk Evaluation Tool (CARET) [27]. The remaining 14 studies applied diverse criteria to categorize alcohol consumption, including definitions of moderate, excessive, heavy, and binge drinking [24,26,30,32,34-40,42,44,45]. However, one study did not specify its measurement unit [43] (Table 2).

**Table 2:** Pooled effects of community-based interventions on binary and continuous alcohol use measures. ^a^, pooled odds ratio after exposure to intervention; ^b^= pooled standardized mean difference; above moderate level alcohol consumption, more than two drinks per day for men and more than one drink for women; CI, confidence interval; I^2^, describes the percentage of variation across studies due to heterogeneity.

Outcome Measures	Number of Studies	Effect Size (95%CI)	I^2^ (%)
Proportion of low-risk alcohol consumption	3	1.99^ a^ (1.04, 3.81)	30%
Proportion of above moderate level alcohol consumption	3	0.50^a^ (0.37, 0.68)	0%
Drinks per week (continuous)	3	-0.08^b^ (-0.14, -0.03)	0%

Types of CBIs

**Figure 4:**
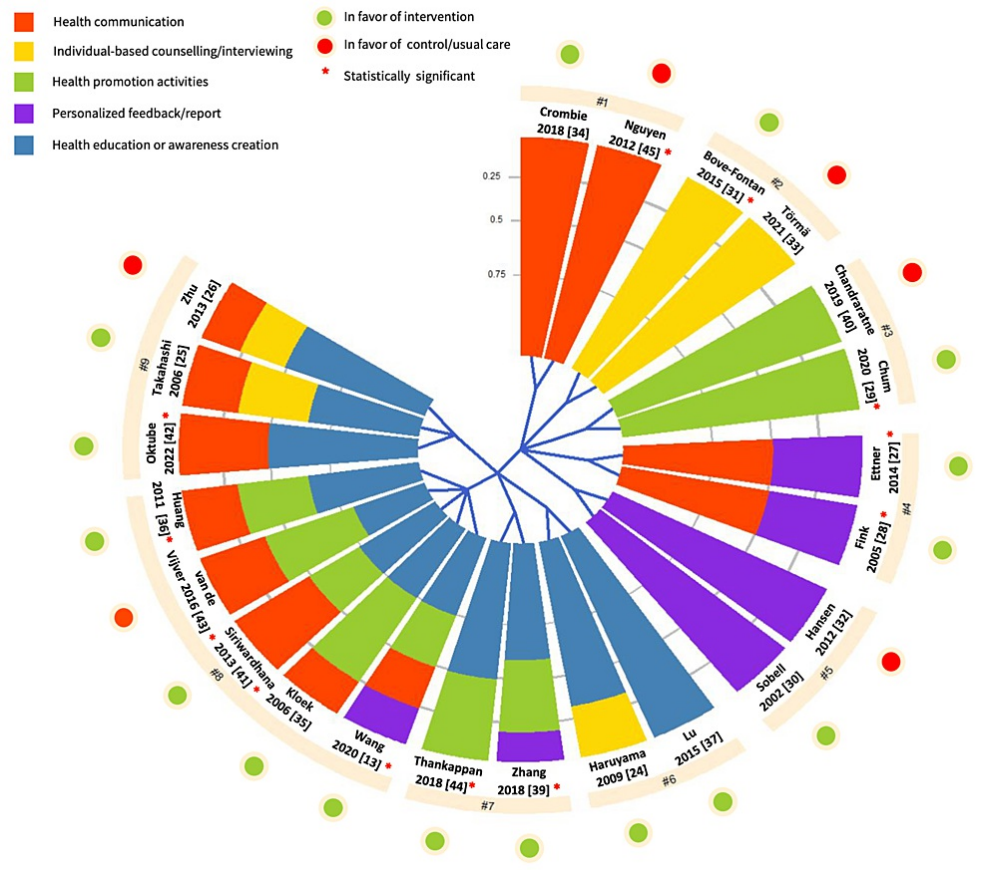
Hierarchical clustering analysis of 22 community-based interventions based on five intervention strategies. Each slice of the chart represents the study intervention(s). The sectors in each chart indicate what types of domains were included in each study, with the area of each sector corresponding to the proportion of each intervention type within one study. Meanwhile, the blue branches starting from the center of the chart show how the interventions were divided into the three main clusters with nine sub-nodes, indicating that the study in each cluster has a more similar domain profile compared to the study in other clusters. Health communication interventions refer to the use of posters, leaflets, newsletters, booklets, tip sheets, text messages, newspapers, media, and pamphlets, delivered electronically or in print. Individual-based counseling includes motivational interviewing, either face-to-face or by phone. Health promotion activities refer to youth agents of change, workplace wellness initiatives, support groups, housing rent supplements, training healthcare staff, and initiatives to improve free access to healthcare and facilities promoting a healthy lifestyle. Personalized feedback/report, an individualized feedback or report about participants’ alcohol consumption, sent electronically or by mail. Health education and awareness creation intervention refers to lectures, sessions, workshops, street dramas, and demonstrations, delivered face-to-face or via phone.

Interventions reviewed encompassed a mix of primordial and primary prevention strategies, based in community settings (n=16) [25-27,29,32,33,35-38,40-45], primary healthcare (n=4) [27,28,31,39], or homes [30,34] (Table 1). Strategies included health education and awareness programs employing individual or group-based methods for lectures, sessions, workshops, street dramas, and demonstrations, delivered face-to-face or via phone [24-26,35-39,41-44]. Health promotion activities/services featured youth agents of change [40], workplace wellness initiatives [38], support groups [39,43,44], housing rent supplements [29], training healthcare staff [36], and initiatives to improve free access to healthcare [29,41,43] and facilities promoting a healthy lifestyle [35]. Additionally, interventions provided individual-based counseling and motivational interviewing, either face-to-face or by phone [24-26,31,33]. Health communication was utilized in 12 studies through posters, leaflets, newsletters, booklets, tipsheets, text messages, newspapers, media, and pamphlets, delivered electronically or in print [25-28,34-36,38,41-43,45]. Personalized reports or feedback on participants’ alcohol consumption, sent electronically or by mail, were also utilized [27,28,30,32,38,39] (Figure 4).

The majority (n=13) of the studies employed multicomponent interventions (combination of two or more CVD prevention strategies) [24-28,35,36,38,39,41-44], whereas nine studies applied single-component intervention [29-34,37,40,45] (Figure 4). Most of the interventions targeted high-risk groups (n=15), including people who are obese [26,42], hypertensive [26,37,38,45], dyslipidaemic [26,31], diabetic [26,44], older adults [28,39], socially disadvantaged [34,35,43], alcohol abusers [30], mentally-ill [29], heavy drinkers [32], at-risk drinkers [27], and/or had at least two CVD risk factors [42] (Table 3).

**Table 3:** Summary findings of the effectiveness of community-based interventions in alcohol consumption. †, Neighborhood health work; Mean difference, compares the change in outcomes from pre-intervention to post-intervention between two groups; Adjusted odds ratio (after intervention); 95%CI, 95% confidence interval (two-sided); %, Percentage; I, demonstrated reduced alcohol consumption in favor of the intervention group; C, demonstrated reduced alcohol consumption in favor of the usual care or attention control group; *, statistically significant with p-values of less than 0.05 and/or 95%CIs excluding the null values (post-intervention AOR=1/mean difference=0); ^R^, range of effect sizes in the intervention group reported in year 1994, 1999, 2004, 2009, and 2014; ^a^, intervention group (Västerbotten); ^b^, control group (Norbotten)

Study ID | Country	Study design	Intervention	Comparison	Target group	Measured alcohol consumption outcome	Effect measure	Direction	Effect size	95% CI	p-value
Siriwardhana et al., 2013 [41] | Sri Lanka	Cluster- Randomized controlled trial	Multicomponent educational interventional program (street drama, poster campaign, leaflet, brief intervention)	Brief Intervention	All male adults	Low risk level of drinking (Alcohol Use Disorder Identification Test score 0-7)	Adjusted Odds Ratio	I	2.88*	1.57, 5.28	Not reported
Takahashi et al., 2006 [25] | Japan	Randomized controlled trial	Moderate-intensity dietary counselling, lecture	Usual care	Free-living healthy subjects	Alcohol use (grams/ day)	Mean difference	I	-9.10	-42.9, 3.7	Not reported
Lu et al., 2015 [37] | China	Randomized controlled trial (3-arm)	Regular lecture	Self- learning reading	Hypertensive patients (low socioeconomic status)	Current alcohol drinkers (drank alcohol at least once/week over 6 months)	Adjusted Odds Ratio	I	0.49	0.16, 1.47	Not reported
		Interactive workshop	Self- learning reading	Hypertensive patients (low socioeconomic status)	Current alcohol drinkers (drank alcohol at least once/week over 6 months)	Adjusted Odds Ratio	I	0.99	0.39, 2.47	Not reported
		Regular lecture	Self- learning reading	Hypertensive patients (low socioeconomicstatus)	Alcohol consumption (grams/day)	Mean difference	I	-0.7	-30.08, 31.48	Not reported
		Interactive workshop	Self- learning reading	Hypertensive patients (low socioeconomic status)	Alcohol consumption (grams/day)	Mean difference	I	-18.3	-26.46, 63.06	Not reported
Ettner et al., 2014 [27] | United States	Cluster- Randomized controlled trial	Educational intervention (educational booklet)	Usual care	At-risk drinkers	Low risk level of drinking (comorbidity alcohol risk evaluation tool)	Adjusted odds ratio	I	2.019*	1.59, 2.57	Not reported
		Educational intervention (educational booklet)	Usual care	At-risk drinkers	Alcohol consumption (Drinks per week)	Mean difference	I	-1.59*	-2.55, -0.62	<=0.01
Zhang et al., 2018 [39] | China	Randomized controlled trial	Multicomponent intervention (Personalized older-centered Integrated Health Management Model Project)	Usual care	Elders	Moderate Alcohol Use (Alcohol intake less than 350ml per week)	Adjusted odds ratio	I	0.496*	0.37, 0.67	<0.001
Chandraratne et al., 2019 [40] | Sri Lanka	Cluster- Randomized controlled trial	Health promotion strategies & counselling using youths	No intervention	Adults	low risk of drinking level was two drinks/day for men and one drink/day for women	Adjusted odds ratio	C	0.496	0.22,1.13	0.27
Hansen et al., 2012 [32] | Denmark	Randomized controlled trial (3-arm)	Internet-based brief personalized feedback	No intervention	Non-Treatment-Seeking Adult Heavy Drinkers	Binge drinking (Drinking five or more drinks per occasion at least once a week)	Adjusted odds ratio	C	1.066	0.79, 1.44	0.30
		Internet-based brief personalized advice	No intervention	Non-Treatment-Seeking Adult Heavy Drinkers	Binge drinking (Drinking five or more drinks per occasion at least once a week)	Adjusted odds ratio	C	1.108	0.85, 1.51	0.70
		Internet-based brief personalized feedback	No intervention	Non-Treatment-Seeking Adult Heavy Drinkers	Alcohol intake (drinks/week)	Mean difference	I	0.3	-2.25, 2.85	0.72
		Internet-based brief personalized advice	No intervention	Non-Treatment-Seeking Adult Heavy Drinkers	Alcohol intake (drinks/week)	Mean difference	I	0.15	-2.23, 2.53	0.47
Bóveda-Fontán et al., 2015 [31] | Spain	Cluster-Randomized controlled trial	Motivational interview	Standard practice	Primary care patients with uncontrolled dyslipidemia	Alcohol intake (unit of drinks/week)	Mean difference	I	-8.92*	-11.01, -6.84	<0.001
Thankappan et al., 2018 [44] | India	Cluster- Randomized controlled trial	Peer-support lifestyle intervention	Education booklet with lifestyle change advice	High-risk individuals identified based on a simple diabetes risk score	Standard drinks of alcohol (per drinking occasion)	Mean difference	I	-0.044*	-0.08, -0.004	0.03
		Peer-support lifestyle intervention	Education booklet with lifestyle change advice	High-risk individuals identified based on a simple diabetes risk score	Current alcohol use (Consumed an alcoholic drink (spirits, wine, beer, or toddy [palm wine]) in the past 30 day)	Adjusted odds ratio	I	0.77*	0.62, 0.95	0.018
Sobell et al., 2002 [30] | Canada	Randomized controlled trial	Motivational enhancement/ personalized feedback	Bibliotherapy/ drinking guidelines	Alcohol abusers who had never sought help or treatment	% of drinking days in the past year	Adjusted odds ratio	I	0.998	0.72,1.37	Not reported
		Motivational enhancement/ personalized feedback	Bibliotherapy/ drinking guidelines	Alcohol abusers who had never sought help or treatment	Drinks per drinking days in the past year	Mean difference	-	0.00	-0.64, 0.64	Not reported
		Motivational enhancement/ personalized feedback	Bibliotherapy/ drinking guidelines	Alcohol abusers who had never sought help or treatment	Days drinking per week in the past year	Mean difference	I	-0.10	-0.51, 0.31	Not reported
		Motivational enhancement /personalized feedback	Bibliotherapy/drinking guidelines	Alcohol abusers who had never sought help or treatment	Drinks per week in the past year	Mean difference	I			
Crombie et al., 2018 [34] | United Kingdom	Randomized controlled trial	Texting to Reduce Alcohol Misuse	Texts on general health	Socially disadvantaged areas who had two or more episodes of binge drinking (> 8 UK units on a single occasion) in the preceding 28 days	% of men with three or more occasions of binge drinking (> 8 units) in previous 28 days (at 12 months)	Adjusted odds ratio	I	0.79	0.57, 1.08	0.14
		Texting to Reduce Alcohol Misuse	Texts on general health	Socially disadvantaged areas who had two or more episodes of binge drinking (> 8 UK units on a single occasion) in the preceding 28 days	men with three or more occasions of heavy binge drinking (> 16 units) in previous 28 days	Adjusted odds ratio	I	0.97	0.64,1.46	0.87
		Texting to Reduce Alcohol Misuse	Texts on general health	Socially disadvantaged areas who had two or more episodes of binge drinking (> 8 UK units on a single occasion) in the preceding 28 days	% of men AUDIT positive (>7 score) at 12 months	Adjusted odds ratio	C	1.34	0.95, 1.89	0.095
		Texting to Reduce Alcohol Misuse	Texts on general health	Socially disadvantaged areas who had two or more episodes of binge drinking (> 8 UK units on a single occasion) in the preceding 28 days	Total alcohol consumption at 12 months	Mean difference	C	4.46	-11.1, 20.03	0.57
Nguyen et al., 2012 [45] | Vietnam	Non-randomized controlled studies	A hypertensive-targeted management program integrated with a community-targeted health promotion Vs	Conventional healthcare	Hypertensive patients and general population	% of Heavy alcohol consumption (>2 standard unit/day for women and >3 standard unit/day for men)	Adjusted odds ratio	C	1.213*	1.01,1.46	Not reported
Van de Vijver et al., 2016 [43] | Kenya	Non-randomized controlled studies	The multi-component intervention (Raising awareness prior to the door-to-door campaign; Improving access to screening; Facilitating access to treatment; Promoting long-term retention in care)	Access to cardiovascular disease standard of care	Adults aged 35 and above	% of Alcohol use (unspecified)	Adjusted odds ratio	C	1.62*	1.13, 2.30	0.008
Huang et.al., 2011 [36] | China	Non-randomized controlled studies	Comprehensive intervention measures, which included education and behavior and lifestyle guidance	Access to standard health care	Adults aged above 35	% of more than two drinks per day/men & more than one for women	Adjusted odds ratio	I	0.48*	0.36, 0.65	<0.05
Kloek et al., 2006 [35] | Netherlands	Non-randomized controlled studies	Multicomponent Interventions ‘‘Wijkgezondheidswerk’’ †	No intervention	General population in three deprived neighborhoods	Excessive alcohol consumption (six or more glasses on 3 or more days a week or four or more glasses on 5 or more days a week)	Adjusted odds ratio	I	0.54*	0.15, 1.73	Not reported
		Multicomponent intervention ‘‘Wijkgezondheidswerk” †	No intervention	General population in three deprived neighborhoods	Alcohol consumption (glasses/day)	Mean difference	-	0.00	-0.14, 0.14	Not reported
Zhu et al., 2013 [26] | Japan	Non-randomized controlled studies	Individual counselling and group sessions(motivational interviewing, talks, lectures)	No Intervention	Participants with cardiovascular disease risk factors	% Drinking alcohol every day at 18 months	Adjusted odds ratio	C	1.23	0.78, 1.94	Not reported
		Individual counselling and group sessions(motivational interviewing, talks, lectures)	No Intervention	Participants with cardiovascular disease risk factors	% Drinking alcohol less than 22 grams at 18 months	Adjusted odds ratio	C	1.28	0.77, 2.15	Not reported
Fink et al., 2005 [28]| United States	Cluster-randomized controlled trial	Combined report (patients received education and physicians received report of patients drinking)	Usual care	Older patients aged 65 and above	Lower risk drinking (classification using Computerized Alcohol Related Problem Scoring)	Adjusted odds ratio	I	1.22*	1.16, 1.31	Not reported
		Patient report (only patients received education)	Usual care	Older patients aged 65 and above	Lower risk drinking (classification using Computerized Alcohol Related Problem Scoring)	Adjusted odds ratio	I	1.58*	1.47, 1.71	Not reported
		Combined report (patients received education and physicians received report of patients drinking)	Usual care	Older patients aged 65 and above	Decrease in drinks per week	Adjusted regression coefficient	I	1.14*	0.59, 1.69	Not reported
		Patient report (only patients received education)	Usual care	Older patients aged 65 and above	Decrease in drinks per week	Adjusted regression coefficient	C	0.33	-0.18, 0.83	Not reported
Haruyama et al., 2009 [24] | Japan	Non-randomized controlled studies	Multicomponent interventions (counselling, lecture, exercise session, workshop, newsletter)	Usual program (lecture & health newsletter)	General population	Drinking alcohol (<20 grams/day, <6 days/week for both males and females)	Adjusted odds ratio	I	0.48	0.20, 1.18	0.152
Wang et al., 2020 [38] | China	Cluster-randomized controlled trial	Routine care for prevention or treatment of diseases	Workplace wellness program for all employees & guidelines-based hypertension management protocol that focused on hypertensive participants	Work place employees	% of consumption of at least 1 drink per week	Adjusted odds ratio	I	0.66	0.57, 0.77	0.0336
Chum et al., 2020 [29] | Canada	Randomized controlled trial	Housing using rent supplements combined with support service	Treatment as usual were not provided with any active intervention or support	Homeless adults (above 18 years) who had serious mental illness and who resided in the Toronto area	Number of days consumed alcohol to intoxication in the past 30 days	Mean difference	I	-1.58*	-2.88, -0.27	Not reported
Okube et al., 2022 [42] | Kenya	Randomized controlled trial	Individualized health education and recommendations on risk factors for cardiovascular diseases	Routine care provided in the hospital by health care workers as per the conventional clinical practice	Adults with common behavioral risk factors for metabolic syndrome & related cardiovascular diseases	Standard drinks of alcohol (per drinking occasion)	Mean difference	I	-1.61*	-2.22, -1.00	Not reported
		Individualized health education and recommendations on risk factors for cardiovascular diseases	Routine care provided in the hospital by health care workers as per the conventional clinical practice	Adults with common behavioral risk factors for Metabolic syndrome and related cardiovascular diseases	% of more than two drinks per day/ men and more than one for women	Adjusted odds ratio	I	0.64	0.32, 1.28	Not reported
Törmä et al., 2021 [33] | Sweden	Non-randomized controlled studies	Cardiovascular Prevention Program- Individual Health Assessment and Counselling on Healthy Lifestyle and Food Habits	No Cardiovascular Prevention Program	Randomly selected above the age of 40 years Residents in two counties						
Zhu et al., 2013 [26] | Japan	Non-randomized controlled studies	Individual counselling and group sessions(motivational interviewing, talks, lectures)	No Intervention	Participants with cardiovascular disease risk factors	% Drinking alcohol every day at 18 months	Adjusted odds ratio	C	1.23	0.78, 1.94	Not reported
		Individual counselling and group sessions(motivational interviewing, talks, lectures)	No Intervention	Participants with cardiovascular disease risk factors	% Drinking alcohol less than 22 grams at 18 months	Adjusted odds ratio	C	1.28	0.77, 2.15	Not reported
Fink et al., 2005 [28]| United States	Cluster-randomized controlled trial	Combined report (patients received education and physicians received report of patients drinking)	Usual care	Older patients aged 65 and above	Lower risk drinking (classification using Computerized Alcohol Related Problem Scoring)	Adjusted odds ratio	I	1.22*	1.16, 1.31	Not reported
		Patient report (only patients received education)	Usual care	Older patients aged 65 and above	Lower risk drinking (classification using Computerized Alcohol Related Problem Scoring)	Adjusted odds ratio	I	1.58*	1.47, 1.71	Not reported
		Combined report (patients received education and physicians received report of patients drinking)	Usual care	Older patients aged 65 and above	Decrease in drinks per week	Adjusted regression coefficient	I	1.14*	0.59, 1.69	Not reported
		Patient report (only patients received education)	Usual care	Older patients aged 65 and above	Decrease in drinks per week	Adjusted regression coefficient	C	0.33	-0.18, 0.83	Not reported
Haruyama et al., 2009 [24] | Japan	Non-randomized controlled studies	Multicomponent interventions (counselling, lecture, exercise session, workshop, newsletter)	Usual program (lecture & health newsletter)	General population	Drinking alcohol (<20 grams/day, <6 days/week for both males and females)	Adjusted odds ratio	I	0.48	0.20, 1.18	0.152
Wang et al., 2020 [38] | China	Cluster-randomized controlled trial	Routine care for prevention or treatment of diseases	Workplace wellness program for all employees & guidelines-based hypertension management protocol that focused on hypertensive participants	Work place employees	% of consumption of at least 1 drink per week	Adjusted odds ratio	I	0.66	0.57, 0.77	0.0336
Chum et al., 2020 [29] | Canada	Randomized controlled trial	Housing using rent supplements combined with support services	Treatment as usual were not provided with any active intervention or support	Homeless adults (above 18 years) who had serious mental illness and who resided in the Toronto area	Number of days consumed alcohol to intoxication in the past 30 days	Mean difference	I	-1.58*	-2.88, -0.27	Not reported
Okube et al., 2022 [42] | Kenya	Randomized controlled trial	Individualized health education and recommendations on risk factors for cardiovascular diseases	Routine care provided in the hospital by health care workers as per the conventional clinical practice	Adults with common behavioral risk factors for metabolic syndrome & related cardiovascular diseases	Standard drinks of alcohol (per drinking occasion)	Mean difference	I	-1.61*	-2.22, -1.00	Not reported
		Individualized health education and recommendations on risk factors for cardiovascular diseases	Routine care provided in the hospital by health care workers as per the conventional clinical practice	Adults with common behavioral risk factors for Metabolic syndrome and related cardiovascular diseases	% of more than two drinks per day/ men and more than one for women	Adjusted odds ratio	I	0.64	0.32, 1.28	Not reported
Törmä et al., 2021 [33] | Sweden	Non-randomized controlled studies	Cardiovascular Prevention Program- Individual Health Assessment and Counselling on Healthy Lifestyle and Food Habits	No Cardiovascular Prevention Program	Randomly selected above the age of 40 years Residents in two counties	Average estimated percentage of energy from alcohol intake	Mean	C	1.4-2.3 ^R,a^ 1.3-2.2 ^R,b^	Not reported	Not reported

*Narrative Analysis* 

**Figure 5:**
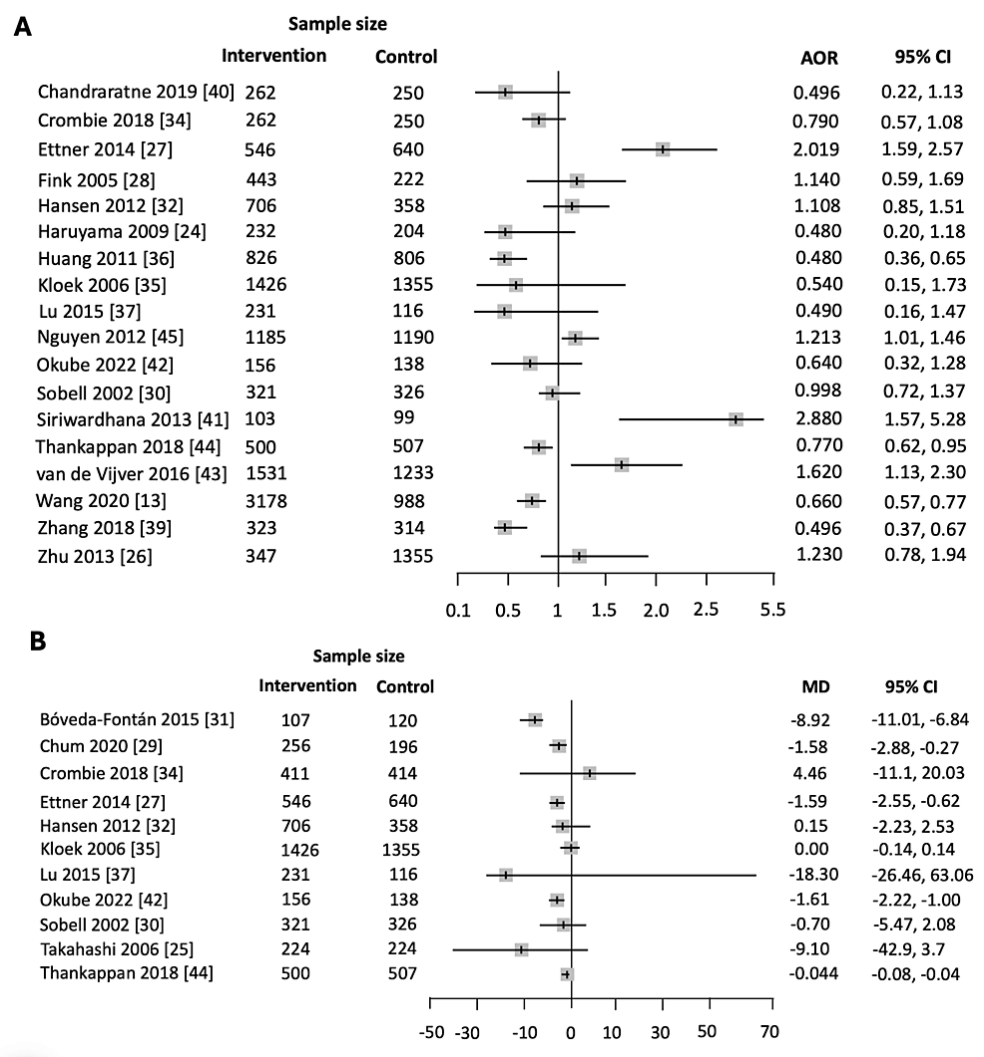
Forest plots for the included studies for studies reporting (A) the (adjusted) odds-ratio after exposure to the intervention and (B) mean difference. AOR, Adjusted odds ratio; MD, Mean difference

A comprehensive summary of the direction of the effects of community-based interventions on alcohol consumption has been provided in Table 3 and Figure 5. In 16 out of the 22 studies, the observed effects favored the intervention group, showing a greater reduction in alcohol consumption compared to the control group. This includes six CRCTs [27,28,31,38,41,44], seven RCTs [25,29,30,34,37,39,42], and three NRCs [24,35,36]. Conversely, in the remaining six studies, comprising one CRCT, one RCT, and four NRCs, the reduction in alcohol consumption was favorable in the control group compared to the intervention group [26,32,33,40,43,45].

Siriwardhana et al. demonstrated the most pronounced effect (AOR=2.88, 95%CI: 1.57, 5.28) favoring the intervention group (multi-component intervention) compared to the control group (brief intervention) [41]. Meanwhile, Sobell et al. reported the lowest effect (AOR=0.998, 95% CI: 0.72, 1.37) favoring the intervention group (motivational enhancement/personalized feedback) compared with the control group (bibliotherapy/drinking guidelines) [30]. When comparing the mean differences in alcohol consumption between the intervention and control groups, Lu et al. reported the most pronounced change in mean reduction of alcohol consumption of 18.3 g per day (95%CI: 26.46, 63.06) in the intervention group (interactive workshop) compared to the control group [37]. Thankappan et al., on the other hand, reported the smallest mean difference with a reduction of only -0.044 standard drinks per drinking occasion (95%CI: -0.08, -0.004) among the intervention group compared to the control [44] (Table 3).

Among the 16 studies for which the effect favored the intervention group compared to the control group, 10 studies were found to be statistically significant [27-29,31,36,38,39,41,42,44] (Table 3). These included six CRCTs [27,28,31,38,41,44], three RCTs [29,39,42], and only one NRC study with a high risk of bias due to confounding [36]. Conversely, out of the six studies where the effect favored the control group over the intervention group, only two NRCs showed statistical significance [43,45]. The remaining 10 studies (one CRCT, five RCTs, and four NRCs) found no statistically significant difference between intervention and control groups [24-26,30,32-35,37,40] (Table 3, Figure 4). 

Among the 10 studies that demonstrated a significant reduction in alcohol consumption favoring the intervention group over the control group, seven studies reported dichotomous outcome measures [27,28,36,38,39,41,44]. Notably, three of these studies employed validated alcohol assessment tools (such as AUDIT, CARET, and CARPS) to assess risky alcohol consumption [27,28,41]. Conversely, among the two NRCs that showed significant effects favoring the control group [43,45], one did not specify the measurement unit for alcohol consumption [43].

Out of the 10 studies that showed a statistically significant reduction in alcohol consumption in the intervention group compared to the control, the majority (n=8) employed multi-component interventions [27,28,36,38,39,41,42,44]. Among these eight multi-component interventions, most (n=6) utilized health education and awareness creation [36,38,39,41,42,44] combined with either health promotion activities [36,38,39,41,44] or health communication messages in printed format [42]. Meanwhile, out of the 10 studies that effectively reduced alcohol consumption in the intervention group, only two studies employed single-component interventions [29,31].

In contrast, among the 10 studies that showed no statistically significant difference between the control and intervention groups, the majority (n=6) employed single-component interventions [30,32-34,37,40]. Two of these studies utilized personalized feedback delivered electronically [30,32], while one each used health education [37], health communication via text messages [34], individual-based counseling [33], and health promotion activities using youth agents of change [40]. Of the two NRCs for which the effect favored the control compared to the intervention group, one utilized health communication delivered through traditional media [45] (Figure 4).

Of the interventions that lasted for 12 months and above (n=12) [28,29,31,35-40,42,44,45], eight studies showed a statistically significant reduction in alcohol consumption in the intervention group [28,29,31,36,38,39,42,44]. In contrast, out of the eight short-term interventions (less than 12 months) [24-26,30,32,34,41,43], most (n=6) did not indicate differences between the intervention and control group (Table 1).

Out of the total 10 studies that were conducted in low-middle income countries [36-45], most (n=6) indicated a significant reduction in alcohol consumption favoring the intervention group [36,38,39,41,42,44]. In contrast, of 12 studies that were conducted in high-income countries, the majority (n=8) found no difference in alcohol consumption between the intervention and control group [24-26,30,32-35] (Table 1).

Meta-Analysis

**Figure 6:**
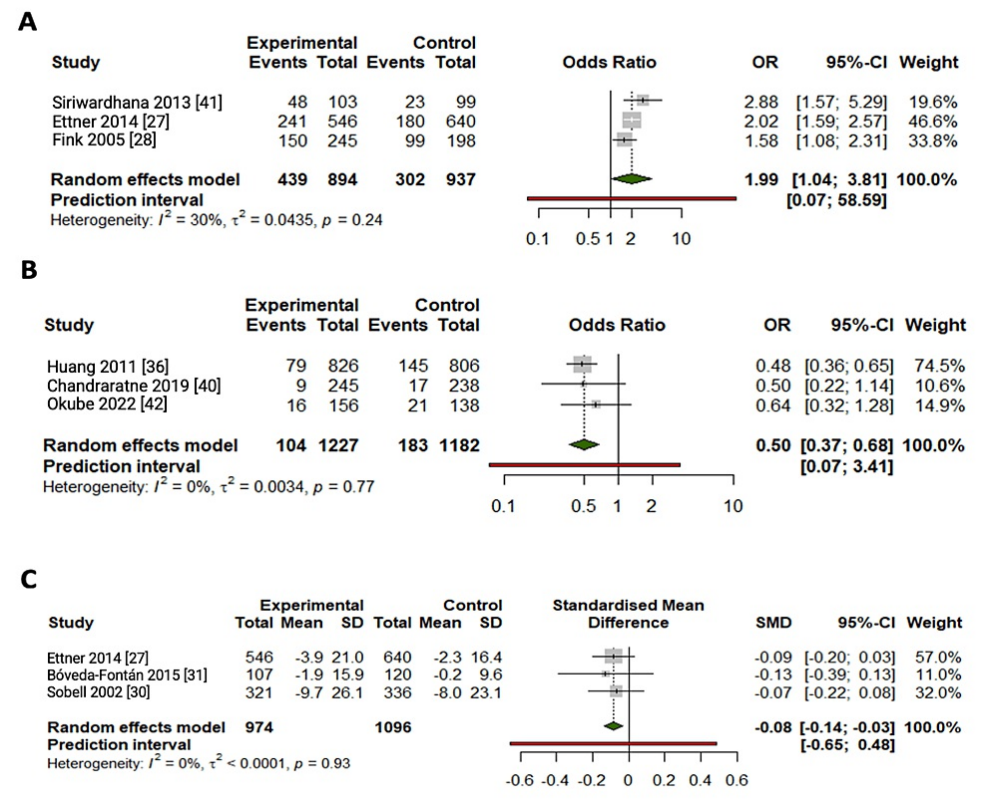
Forest plots for studies reporting (A) the odds ratio of low-risk alcohol consumption measured by validated tools assessing at-risk alcohol consumption and (B) the odds ratio of above moderate-level alcohol consumption, and (C) the mean difference of drinks per week outcome measures at 12 months follow-up. SD, Standard deviation; SMD, Standardized mean difference, OR, Odds ratio; CI, Confidence interval

CBIs were effective in increasing the proportion of participants classified as “low-risk drinkers” in the intervention group as compared to the control group (OR=1.99, 95%CI: 1.04, 3.81). A decrease in the above “moderate level” of alcohol drinking (two drinks per day for men and one drink per day for women) was observed in those who received a CBI as compared to their control counterparts (OR=0.50, 95%CI: 0.37, 0.68). Additionally, a decrease was observed in the number of drinks consumed per week in those who received a CBI as compared to those who were in the control group (Table 2, Figure 6).

Discussion 

The increasing burden of CVDs has spurred the exploration of effective prevention strategies, with CBIs emerging as a pivotal avenue. This review assessed the types and effectiveness of CBIs targeting the reduction of alcohol consumption - a strategic approach aimed at alleviating the CVD burden by targeting one of its main risk factors. We assessed 22 eligible studies by exploring varying intervention components, settings, duration, outcome measures, and their impact on the reduction of alcohol consumption. Due to the heterogeneity in outcome measures, only eight studies with similar measures of alcohol consumption measures were integrated into the meta-analysis. Overall, our findings revealed a reduction in different measures of drinking patterns and risky alcohol consumption within the intervention group in comparison to the control group. Furthermore, multicomponent interventions, especially those that combined health education with health promotion activities, demonstrated a more pronounced effect in the intervention group.

Our findings yielded insights into the effectiveness of CBIs in reducing alcohol consumption. Specifically, we observed a substantial reduction in the number of drinks consumed per week in the intervention group compared to the control at 12-month follow-up. Furthermore, a reduction in alcohol consumption was observed in the intervention group, particularly in terms of above-moderate-level and risky alcohol consumption. These findings underscore the potential of community-based strategies to successfully address alcohol consumption as a contributing risk factor for CVDs.

While these results are promising, it is important to acknowledge the substantial heterogeneity observed in the measures of alcohol consumption across the included studies. This variation reflects the diverse and inconsistent approaches employed in assessing alcohol consumption. Previous reviews have similarly highlighted this issue and emphasized the importance of using validated and consistent assessment measures to enable meaningful comparisons and accurate evaluations of intervention effectiveness [16,46]. The significance of employing consistent and comprehensive assessment tools was also underscored by our findings. The majority of the studies that employed validated and comprehensive tools, such as AUDIT, CARET, and CARPS, to assess unhealthy alcohol consumption demonstrated a finding favoring the intervention group. This could be attributed to these tools capturing a wide range of recommended factors associated with alcohol consumption, including drinking patterns, alcohol-related problems, and high-risk consumption enabling an accurate and comprehensive evaluation of alcohol consumption [46,47]. Therefore, such and other similar assessment tools should be considered for use depending on the aims and context of community-based cardiovascular interventions targeting the reduction of alcohol consumption.

Previous studies have demonstrated the effectiveness of multi-component intervention strategies [15,16], which is consistent with our review. In our review, multi-component interventions that specifically combined health education and awareness creation with health promotion activities exhibited a more pronounced effect in reducing alcohol consumption. Similar results have been reported in previous systematic reviews exploring CBIs targeting alcohol consumption and other major risk factors of CVDs [16,48]. This might be attributed to the fact that such strategies intervene at both the individual level and within the surrounding environment, which is essential for facilitating the desired behavior change. Health education serves to dispel the widespread myth of alcohol consumption benefits and foster an accurate understanding of its harmful effects on cardiovascular health [3,48]. Coupling this foundational element with health promotion activities may not only aid in reinforcing but may also empower individuals to reduce their alcohol consumption. Therefore, the comprehensive and synergistic nature of multi-component interventions, encompassing health education and health promotion activities, is instrumental in fostering successful reductions in alcohol consumption in the context of CVD prevention and should be considered in future interventions.

Furthermore, intervention duration appeared to play a role in determining their effectiveness in reducing alcohol consumption. Most of the longer-term interventions, lasting 12 months or more, demonstrated a reduction in alcohol consumption. Previous studies have also indicated a longer intervention duration to be positively associated with observing strong evidence for an intervention’s effectiveness and better alcohol-related outcomes [16,49,50]. Moreover, a prior study concluded a longer intervention duration (intervention sessions spread over up to 12 months or more) was associated with a higher likelihood of abstaining from consuming alcohol and other drugs after controlling for intensity [51]. Changing and sustaining desired behavior, such as reducing alcohol consumption, requires an extended period to reinforce, practice, and adapt to new habits. Achieving relevant behavior change within a short timeframe may be challenging due to the potential for relapse into previous drinking habits [52]. Thus, the value of persistent efforts to promote behavior change and the challenges of achieving immediate results in the context of alcohol consumption reduction should not be underestimated. Consequently, prioritizing a longer duration of intervention is imperative, besides the intensity of the intervention, when aiming to accurately assess its effectiveness on alcohol consumption.

Notably, the majority of interventions conducted in LMICs demonstrated effectiveness in reducing alcohol consumption, whereas the majority of studies conducted in HICs exhibited non-significant findings. This discrepancy could be attributed to the potential existence of unobserved influence of other public health policies designed for reducing alcohol consumption in addition to the CVD CBIs [53]. This may lead to the underestimation of the intervention’s effect in HICs. Despite the heaviest burden of heavy episodic drinking among both males and females being prevalent in LMICs [54], there exists a disproportionate distribution of community-based CVD interventions between HICs and LMICs. This finding aligns with prior reviews that have underscored the limited presence of community-based CVD interventions, particularly in LMICs, especially in Sub-Saharan Africa [16,17,55]. Thus, there is a critical need to enhance research capacity in LMICs for the implementation of CBIs targeting CVD risk factors, including alcohol consumption. This emphasis is crucial due to the potential cost-effectiveness and the heightened prevalence of heavy episodic drinking within these regions.

Methodological Considerations 

Interestingly, the effectiveness of CBIs varied based on the type of study design. Randomized studies demonstrated reductions in alcohol consumption favoring the intervention group compared with non-randomized studies. Specifically, almost all CRCTs demonstrated reductions in alcohol consumption favoring the intervention group. This disparity in outcomes might be attributed to the inherent design of CRCTs, which account for community-level influences and potentially create a more conducive environment for behavior change through the intervention's spill-over effect, influencing behaviors of participants within the same cluster [56].

However, it is also important to acknowledge the concerns identified in our review regarding the quality of studies assessed using the Cochrane Risk of Bias assessment tool. For the NRCs, biases arising from deviations from intended interventions, confounding, and missing data handling in most studies compromised their quality. Specifically, the two NRC studies that favored the control group in reducing alcohol consumption exhibited issues related to bias due to confounding, selection of participants, classification of intervention, deviation from intended interventions, and missing data handling [43,45]. On the other hand, for randomized studies, deviations from intended interventions mainly affected their quality. Therefore, based on these findings, it is recommended that researchers aiming to implement CBIs targeting alcohol consumption reduction prioritize rigorous study designs, such as CRCTs, which can account for community-level influences, and ensure strict adherence to intervention protocols to minimize deviations and enhance the overall quality of the research.

This review presented the effectiveness of various types of CBIs for reducing alcohol consumption in the context of CVD prevention. Specifically, it underscored the effectiveness of multi-component interventions, particularly those that combine health education with promotion activities. Furthermore, it identified essential components of interventions, offering valuable insights for future researchers to consider, including intervention duration and the utilization of validated assessment tools to comprehensively and accurately measure alcohol consumption outcomes. Lastly, this review emphasized the critical need to enhance research capacity and implement context-specific interventions in LMICs. As such, the findings not only contribute additional evidence for policymakers and public health practitioners but also provide actionable recommendations to strengthen CBIs for CVD prevention with a focus on alcohol consumption reduction.

Limitations

Our review has limitations that should be acknowledged and considered when interpreting its findings. Firstly, language bias may have arisen due to the restriction of articles to the English language. This might lead to a biased understanding of the effects of interventions, as valuable findings from non-English sources are overlooked. Consequently, this might result in an overestimation of the interventions' effectiveness in regions primarily publishing in English while potentially ignoring successful strategies documented in other languages. Secondly, the heterogeneity in measurement approaches used across the included studies precluded meta-analysis for certain outcomes, resulting in difficulties in comparing and synthesizing results. This limitation restricts our ability to pool findings and draw robust conclusions about the effectiveness of CBIs in reducing alcohol consumption. Nevertheless, outcomes not included in the meta-analysis were summarized using narrative synthesis. Lastly, the inclusion of only a small number of studies in the meta-analyses led to wide confidence intervals for the effect sizes, indicating less precise estimates.

## Conclusions

In summary, this review provided substantial evidence of the effectiveness of CBIs targeting the reduction of alcohol consumption as a strategy to mitigate the burden of CVDs. The review included a diverse range of study designs, intervention components, and settings, revealing reductions in alcohol consumption within the intervention groups. Notably, multi-component interventions, particularly those integrating health education and promotion activities, displayed a more pronounced effect in reducing alcohol consumption. The findings underscore the potential of community-based strategies in addressing alcohol consumption as a risk factor for CVDs. Therefore, to enhance the effectiveness of community-based CVD preventive interventions in reducing alcohol consumption, integrating multi-component intervention, and extending the duration of these programs is recommended. Future research should employ standardized and validated tools to measure alcohol consumption outcomes, enhancing the consistency and comparability of results. Furthermore, detailed methodologies and assessments included in the main text enhance the transparency of the review process. Relocating these details might give the impression that certain aspects of the review process are being obscured or de-emphasized.

## Figures and Tables

**Figure 7:**
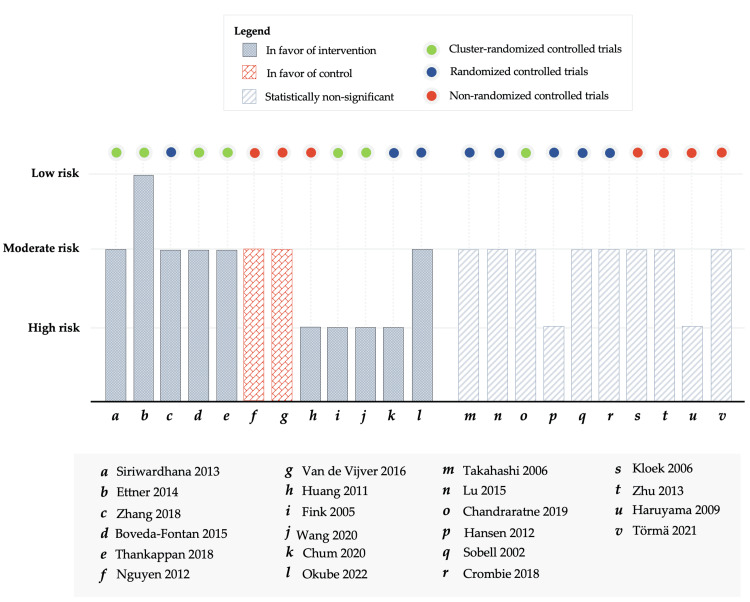
Harvest plot for all included studies by overall risk of bias, effectiveness of the intervention, and their study designs.

## Appendices

**Table 4:** Detailed description of the data extraction form.

Variables	Description
Author and Year	Reference for the study to ensure proper citation and identification.
Follow-Up Duration (FU)	Duration over which the participants were followed to assess the outcomes of the intervention.
Intervention Duration	Length of time the intervention was actively administered.
Risk of Bias (RoB)	Assessment of potential bias in the study's execution, categorized by levels of concern. Data extracted to assess the risk of bias was based on the Cochrane tool for randomized studies (RoB2) and the Risk of Bias in Non-Randomized Studies - of Interventions (ROBINS-I) tool for non-randomized studies.
Context	Rural, Urban, or Mixed; specifying the environment in which the study was conducted.
Setting	Description of the setting such as community-based, primary care, or home-based, specifying where the intervention took place.
Target Group	Characteristics of the population targeted by the study (e.g., age group, specific patient demographics like hypertensive patients, or risk-related characteristics like smokers).
Participants’ Sex	Participant gender distribution within the study.
Outcome Measures	Specific outcomes measured in the study, particularly related to alcohol consumption (e.g., units of alcohol per week, AUDIT scores).
Design	Type of study design utilized, such as RCT (Randomized Controlled Trial), NRCT (Non-Randomized Controlled Trial).
Effect Estimates	Quantitative results from the study, such as mean differences, odds ratios, and other statistical measures.
Outcomes Measures	health outcomes measured by the studies, including factors like Alcohol consumption, blood pressure, dietary intake, or physical activity.
Age of Participants	The age range or average age of participants in the study.
Attrition Rate	The percentage of participants who dropped out of the study.
Sample Size	The total number of participants in the study.
Statistical Methods	The statistical methods used to analyze the data.
Funding Sources	The source of funding for the study.

**Table 5:** Overview of study characteristics and the summary of their findings. *NI, Not indicated; MA, Included in the meta-analysis; OR, Odds ratio; AOR, Adjusted odds ratio; CI, Confidence interval

Author, Year	Intervention Duration (Months)	Intervention Setting	Participant Age Range or Mean (SD)	Sample Size		Summary Finding
				Intervention group (s)	Control group	
Individual randomized studies						
Crombie et al., 2018 [34], United Kingdom	3	Home-based	25-44	411	414	Formal analysis showed that there was no evidence that the intervention was effective [OR = 0.79, 95% CI = 0.57, 1.08; absolute reduction 5.7%, 95% CI = 13.3, 1.9]. The Bayes factor for this outcome was 1.3, confirming that the results were inconclusive.
Hansen et al., 2012 [32], Denmark	6	Community-based	49-66	706	358	Non-significant Intervention effect of the Internet-based brief personalized feedback intervention & Internet-based personalized brief advice, compared with the control group, was –1.4 drinks/week (95% CI =-3.4, 0.6) and -1.2 drinks/week (95% CI= -3.3, 0.9) at 12 months, respectively.
Lu et al., 2015 [37], China	24	Community-based	40-75	231	116	The percentages of current smokers and alcohol drinkers did not change significantly in all three health education intervention groups.
Sobell et al., 2002 [30], Canada ^MA ^	4-8	Home-based	18+	321	326	No significant difference was found between Motivational enhancement/personalized feedback and Bibliotherapy/drinking guidelines.
Takahashi et al., 2006 [25], Japan	2	Community-based	40-69	224	224	No significant intervention effect in terms of alcohol use (gram/day) at the one-year follow-up.
Zhang et al., 2018 [39], China	24	Primary healthcare setting	60+	323	314	A significant intervention effect was observed on moderate alcohol use (less than 350 ml) (0.496, 95% CI=0.367, 0.670).
Chum et al., 2020 [29], Canada	24	Community-based	18+	256	196	Limited evidence suggests that the intervention may reduce daily intoxication by 1.58 (95% CI= -2.88, -0.27) at a greater rate compared with treatment over 24 months.
Okube et al., 2022 [42], Kenya ^MA^	12	Community-based	18-64	156	138	Intake of alcohol significantly (*p* <0.05) declined in the intervention compared to controls by the end-line.
Clustered randomized studies						
Boveda- Fontan et al., 2015 [31], Spain ^MA^	12	Primary healthcare setting	40-75	107	120	The motivational interviewing-based approach led to a significant reduction of 8.92 units/week (95%CI: -6.84, -11.01, *p *<0.001) in the experimental group compared to the control group.
Chandraratne et al., 2019 [40], Sri-lanka ^MA^	12	Community-based	IG-46.1(8.1), CG-44.8(8.2) (Adults)	262	250	No significant intervention effect on low-risk alcohol drinking level (two drinks/day for men and one drink/day for women) was observed at 12-month follow-up.
Ettner et al., 2014 [27], United States ^MA^	NI*	Primary healthcare setting	60+	546	640	At 12 months, the intervention was significantly associated with reductions in at-risk drinking (56% versus 67%; *p *<0.01) and alcohol consumption (-2.19 drinks per week; *p *<0.01)
Fink et al., 2005 [28], United States ^MA^	30	Primary healthcare setting	65+	443	222	The interventions (patient report and combined report) were each associated with greater odds of lower-risk drinking at follow-up than usual care (OR = 1.59 and 1.23, respectively, *p* <.05 for each).
Siriwardhana et al., 2013 [41], Sri-lanka ^MA^	3	Community-based	18-80	103	99	A significant reduction in at-risk drinking in terms of the Alcohol Use Disorder Identification Test (AUDIT) scores in the intervention village compared with the control at 6 and 24 months (*p* < 0.0001)
Thankappan et al., 2018 [44], India	12	Community based	30-60	500	507	At 24 months, compared with the control group, intervention participants had a greater reduction in alcohol use (proportion of those who drank an alcoholic drink in the past 30 days) (RR 0.77, *p* = 0.018).
Wang et al., 2020 [13], China	24	Community-based	18-60	3178	988	At 24 months, compared with the control group, a significant reduction in current drinking (consumption of at least 1 drink per week) was reported in the intervention group (OR=−18.4%; 95% CI, −20.6% to −16.2%; *p* < .001).
Non-randomized studies						
Haruyama et al., 2009 [24], Japan	6	Community-based	65+	232	204	There was no significant difference between intervention and control groups (AOR= 0.485; 95%CI=0.199, 1.18) in terms of drinking alcohol (< 20 grams/day,< 6 days/week) in both males and females.
Huang et al., 2011 [36], China ^MA^	36	Community-based	35+	826	806	The participants in the intervention group exhibited a significant reduction in alcohol consumption (4.2% of participants), after 3 years in comparison with those in the control group with 7.3% increase regular alcohol drinking (more than two drinks per day for men and >= one for women).
Kloek et al., 2006 [35], Netherlands	24	Community-based	18-65	1426	1355	There was no significant impact on alcohol consumption when comparing the intervention neighborhoods (OR=0.96, 95% CI=0.69, 1.33) to the control neighborhoods (OR=0.90, 95% CI=0.70, 1.15).
Nguyen et al., 2007 [45], Vietnam	36	Community-based	25+	1185	1190	There was a significant reduction in alcohol use in the control group (AOR=1.213; 95% CI=1.01, 1.46) compared to the intervention group.
van de Vijver et al., 2016 [43], Kenya	6	Community-based	35+	1531	1233	There was a significant reduction in alcohol use at the population level in the control group (OR=0.71; 95% CI=0.57,0.88) compared to the intervention group.
Zhu et al., 2013 [26], Japan	6	Community-based	40-74	347	1636	No significant difference was observed between the intervention and control group in terms of moderate alcohol use.
Törmä et al., 2021 [33], Sweden	NI*	Community-based	40+	2555	2845	No differences in temporal trend for the estimated percentage of energy intake from alcohol were observed.

**Table 6:** Synthesis Without Meta-Analysis (SWiM) checklist.

SWiM is intended to complement and be used as an extension to PRISMA		
SWiM reporting item	Item description	Page in manuscript where item is reported
1 Grouping studies for synthesis	1a) Provide a description of, and rationale for, the groups used in the synthesis (e.g., groupings of populations, interventions, outcomes, study design)	6,7
	1b) Detail and provide rationale for any changes made subsequent to the protocol in the groups used in the synthesis	7
2 Describe the standardised metric and transformation methods used	Describe the standardised metric for each outcome. Explain why the metric(s) was chosen, and describe any methods used to transform the intervention effects, as reported in the study, to the standardised metric, citing any methodological guidance consulted	6,7
3 Describe the synthesis methods	Describe and justify the methods used to synthesise the effects for each outcome when it was not possible to undertake a meta-analysis of effect estimates	6
4 Criteria used to prioritise results for summary and synthesis	Where applicable, provide the criteria used, with supporting justification, to select the particular studies, or a particular study, for the main synthesis or to draw conclusions from the synthesis (e.g., based on study design, risk of bias assessments, directness in relation to the review question)	6
5 Investigation of heterogeneity in reported effects	State the method(s) used to examine heterogeneity in reported effects when it was not possible to undertake a meta-analysis of effect estimates and its extensions to investigate heterogeneity	6,7
6 Certainty of evidence	Describe the methods used to assess certainty of the synthesis findings	6
7 Data presentation methods	Describe the graphical and tabular methods used to present the effects (e.g., tables, forest plots, harvest plots). Specify key study characteristics (e.g., study design, risk of bias) used to order the studies, in the text and any tables or graphs, clearly referencing the studies included	6
8 Reporting results	For each comparison and outcome, provide a description of the synthesised findings, and the certainty of the findings. Describe the result in language that is consistent with the question the synthesis addresses, and indicate which studies contribute to the synthesis	10-12
Discussion		
9 Limitations of the synthesis	Report the limitations of the synthesis methods used and/or the groupings used in the synthesis, and how these affect the conclusions that can be drawn in relation to the original review question	15,16

**Table 7:** Search strategy used in the Medline database.

#1	( “Community” OR “community-based intervention” OR “community-based” OR “community based” OR “community intervention” OR “population-based intervention” OR “population based” OR “population intervention” OR “community health” OR “community organisation” OR “community organization” OR “community program*” OR “Community level” OR “Community networks” OR “community health services” OR “home based” OR “community participation” OR “community-based research”)
#2	( Interven* OR strateg* OR approach* OR program* OR “health education” OR “health educ*” OR advise OR “raising awareness” OR counsel* OR “health promotion” OR “health campaign” OR “wellness program*” OR “mass media” OR “behaviour* change” OR “behavior* change” OR “lifestyle intervention” OR “lifestyle program*” OR “screening” “motivational interviewing” OR “risk scoring” OR refer* OR training OR “capacity building” OR “peer” OR “peer group” OR “community health worker” OR “CHW” OR “community health volunteer” OR “health worker*” OR “Community Health Extension Worker” OR “Health promoter” OR “Community Health Care Provider” OR “social support” OR “adherence support” OR “coaching” OR “self management” OR self-management OR “outreach” OR “home visit” OR “appointment reminders” )
#3	( “Cardiovascular disease” OR “CVD” OR “CVD risk” OR “cardiovascular disease prevention” OR “cardiovascular disease control” OR “stroke” OR “coronary heart disease” OR “heart diseas*” OR “heart failure” OR “kidney disease” OR “Cardiovascular risk factor” OR “hypertension” OR “raised blood pressure” OR diabetes OR “raised blood sugar” OR “cholest*” OR triglyceride OR HDL OR LDL OR “lipid profile” OR “metabolic syndrome” OR “body mass index” OR “BMI” OR “Overweight” OR “obesity” OR “obese” OR “waist circumference” OR “life style” OR “lifestyle” OR “alcohol” OR “tobacco” OR “smoking” OR “diet*” OR “nutrition” OR “food habit” OR “junk food” OR “fast food” OR “fruit” OR “vegetables” OR “five a day” OR “salt reduction” OR “physical inactivity” OR “physical activity” OR “exercise” OR “stress”)
#4	(“randomized controlled trial” OR “randomized” OR “randomised” OR “controlled study” OR trial OR RCT OR cluster OR CRT OR “comparative study” OR “quasi experimental study” OR “quasi-experiment” OR “experimental” OR “control group” OR “follow up” OR “prospective” or “retrospective” OR placebo OR random* OR “follow-up” OR “non-random*” OR “nonrandom*” OR “before after stud*” OR "before and after" or “time series” or “time-series” OR “interrupted time series” OR longitud* OR “controlled before” OR “pre-post” OR pretest OR posttest OR “pre intervention” or “post intervention”)
#4	#1 AND #2 AND #3 AND #4
#5	Filters: year of publication: (January 2000 to June 2019), Language: English Age: adults (18 and above) population: humans
